# Mechanism, Kinetics and Modelling of Phenol Carboxylation Reactions with CO_2_

**DOI:** 10.3390/ijms252312923

**Published:** 2024-12-01

**Authors:** Aleksa Kojčinović, Blaž Likozar, Miha Grilc

**Affiliations:** 1 Department of Catalysis and Chemical Reaction Engineering, National Institute of Chemistry, Hajdrihova 19, 1000 Ljubljana, Slovenia; aleksa.kojcinovic@ki.si; 2 Graduate School, University of Nova Gorica, Vipavska Cesta 13, 5000 Nova Gorica, Slovenia; 3 Pulp and Paper Institute, Bogišićeva 8, 1000 Ljubljana, Slovenia; 4 Faculty of Polymer Technology, Ozare 19, SI-2380 Slovenj Gradec, Slovenia

**Keywords:** carboxylation, C-C coupling, lignin-derived aromatics, phenol, kinetic modeling

## Abstract

Combining carboxylation reactions using carbon dioxide (CO_2_) as a reactant with phenol results in creation of new C-C bonds, and represents one of the most promising routes in sustainable utilization of ubiquitous and readily available resources for production of highly valuable products. This study provides a detailed and well-structured investigation of the effect of various reaction conditions (reactant loading, reaction duration, temperature, CO_2_ pressure) on the carboxylation of phenol. Sodium phenoxide carboxylation showed well-resolved trends with variation of temperature and time, and resulted in production of salicylic acid (SA) in the range of 11.4 to 47.8%, 4-hydoxybenzoic acid (4HBA) in the range of 2.0 to 8.2%, while the dicarboxylated 4-hydroxyisophthalic acid (4HiPh) was only detected in trace amounts. The effect of the variation of reactant content was shown to be significantly influenced by the reactor size, solid/vessel and gas/solid contact area, as well as the efficiency of the stirring. CO_2_ pressure was shown to be a crucial element, where reactions carried out below 2 MPa CO_2_ did not show any activity. While investigating the reaction mechanism, it was shown that the salt analogues of potential products could be acidified in situ by the moisture present, and immediately degraded back to phenol, thus lowering yields of potentially obtained products. The experimental results were successfully used to compose a kinetic model, which very well describes the experimentally obtained results. As such, this study provides a valuable dataset for valorization of lignocellulosic aromatic compounds as well as highly abundant and environmentally detrimental carbon dioxide into industrially valuable mono- and dicarboxylic acids.

## 1. Introduction

Global warming, as one of the crucial problems in the last couple of decades, continually and increasingly poses threats of possibly irreversible environmental issues. As such, it gives an incentive for thorough investigation of its origins, alleviation of causes, and amelioration of consequences. Carbon dioxide (CO_2_), as a greenhouse gas, contributes greatly to the issue of global warming, and through various Carbon Capture and Utilization (CCU) practices, attempts have been made to lower its negative environmental impact, while simultaneously providing benefits to industry and modern lifestyle [1,2,3].

Biomass valorization has also been identified as a very promising alternative for the utilization of the depleting and environmentally harmful crude oil. Lignocellulosic biomass, and more specifically lignin, represents an abundant and currently poorly valorized source of various aromatic hydrocarbons, among which phenol is one of the most represented compounds, with abundant upgrading potential [4]. These biomass-derived aromatic hydrocarbons show great potential in replacing numerous oil-derived compounds, and are of great value to various industries [5]. The research and industrial communities have identified carbon fixation reactions which represent highly advantageous routes for both CO_2_ and biomass utilization, due to the formation of new C-C bonds and their higher energy density [6]. Such reaction routes lead to the formation of carboxylic acids, a large group of chemical compounds, which due to their abundant physico-chemical properties find roles in various industries. Salicylic acid, a representative of carboxylic acids and more specifically hydroxybenzoic acids, is a highly important chemical which finds roles in industries such as cosmetic, textile, colorings and dyes, and most importantly fine chemicals and the pharmaceutical industries [7,8].

The preparation of salicylic acid is a procedure known for over a century, and nowadays is industrially used as a precursor of the highly important medicine aspirin. The first successful synthesis of salicylic acid was reported in 1860 by Kolbe, who prepared it from carboxylation of dry sodium phenoxide at elevated temperatures [9]; however, Schmitt significantly increased the yields by conducting the reaction under higher pressures of CO_2_ [10], and nowadays, any carboxylation reaction of aromatic hydroxides is called a Kolbe–Schmitt type reaction. The original Kolbe–Schmitt is a two-step reaction, where in the first step phenol reacts with alkali metal oxide or carbonate and forms a metal phenoxide, which then in the second step, under high temperatures and CO_2_ pressures, gets converted to metal salicylate, and lastly gets acidified to salicylic acid [11]. A slight modification of the Kolbe–Schmitt reaction is the Marasse method, in which the phenol, the base, and CO_2_ are introduced directly into the reactor, thus making it a two-step, one-pot reaction [12]. Regardless of the method used, the detailed reaction mechanism is still a topic of ambiguity and great discussion in the literature. Most of the currently available literature focuses on in silico studies, which generally lack thorough experimental work supporting the theoretical findings. Markovic et al. suggested that although the reaction between CO_2_ and sodium phenoxide can take place either on the benzene ring or the polarized O-Na bond, the reaction will proceed with the latter, due to CO_2_ being a weak electrophile. In doing so, it will go through three transition states and three intermediates, with the last step of forming the sodium salicylate being the 1,3 proton shift from C to O atom [13]. Besides salicylic acid, 4-hydroxybenzoic acid (4HBA) was also shown to be a possible product, and various studies have shown that the choice of metal has significant effect on the ratio between SA and 4HBA and their selectivities. Both experimental and theoretical studies have shown that higher atomic radius of the chosen alkali metal leads to the formation of the para-hydroxyl acid [14,15].

Alternative Kolbe–Schmitt type reactions have also been investigated and show quite diversified potential. Plasch et al. [16] showed potential enzymatic carboxylation of resorcinol, Sadamitsu et al. [17] investigated the effect of various bases and organic solvents on resorcinol carboxylation, and Baine et al. [18] showed carboxylation of catechol. Luo et al. [19] investigated carboxylation of various aromatic hydroxides with NaH as a base and trimethyl phenol as an additive under atmospheric pressure. Iijima et al. [20] were the only ones to carry out a systematic study investigating the effect of reaction conditions onto phenol carboxylation. However, they conducted their studies under supercritical CO_2_ and obtained increasing sodium phenoxide (PhONa) conversion (up to 60%) and high SA selectivities. However, the authors failed to show the efficiency of sodium phenoxide preparation and its potential effect on the calculations of conversion and selectivity.

The aim of this study was to construct a well-structured and systematic study of the effect of various reaction conditions on the conversion and selectivity of sodium and potassium phenoxides, as great representatives of lignin-derived compounds, and their activity with gaseous CO_2_, as one of the greatest contributors to environmental issues. To overcome potential complications, ambiguities, and potential inaccuracies with the first step of this Kolbe–Schmitt reaction, i.e., preparation of metal phenoxides, this study will only investigate the reactivity of commercially available sodium and potassium phenoxides. The effect of reaction temperature, reaction duration, substrate loading, and CO_2_ pressure will be studied in a closed batch reactor system. The conversion was first assessed over a wide temperature range (373–523 K), and once the optimal activity was identified, finer temperature steps (5–15 K) were used to monitor the conversion and selectivity changes. To preserve the simplicity of the system, the CO_2_ pressure was investigated up to 3 MPa, as higher pressures would involve supercritical CO_2_ conditions. The reaction time (1, 2, 3 h) and loading (0.15, 0.3, and 0.6 g) were investigated at central temperatures and pressure, which were previously established. All the experimental results obtained from the investigation of effect of the above-mentioned reaction parameters were used to construct the kinetic model, which will describe all the reactions taking place. The results of this study will for the first time provide a single assembly of the effect of the various and most essential reaction conditions on the conversion and selectivity of the highly valuable sodium and potassium phenoxide carboxylation, and provide significant experimental information for their future studies, as well as, for the first time, providing experimental data explaining observed reaction routes, complemented with the kinetic model as an additional confirmation.

## 2. Results

### 2.1. Effect of Reaction Temperature

Primarily, the effect of the reaction temperature on the conversion and selectivity of commercial sodium and potassium phenoxide was carried out in a Parr 2500 reactor system, with three 5 mL parallel batch reactors. The reactions were carried out under 3 MPa of CO_2_ pressure, with the phenoxide loading of 0.3 g, reaction times of 1, 2, and 3 h and various reaction temperatures. At first, a wide range of reaction temperatures was tested to identify the region where the conversion takes place. Sodium phenoxide reactions carried out below 363 K did not show any conversion, while for potassium phenoxide that was the case for reactions below 473 K.

**Figure 1:**
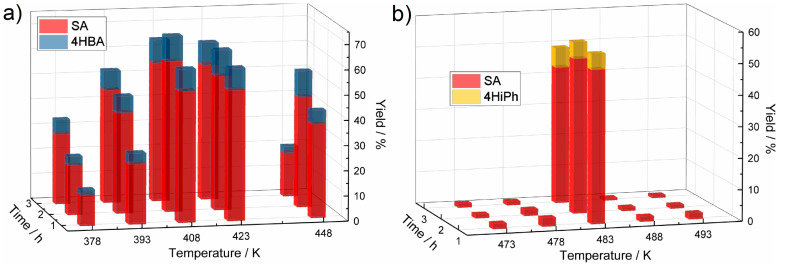
The effect of various temperatures and reaction times on carboxylation of sodium phenoxide (**a**) and potassium phenoxide (**b**).

Figure 1a shows yields of products obtained from sodium phenoxide carboxylation, while Figure 1b shows yields of products from CO_2_ fixation onto potassium phenoxide. Carboxylation of potassium phenoxide, shown in Figure 1b, shows significant formation of carboxylated products, carried out at 483 K and after 1, 2, and 3 h of reaction time. However, the reactions carried out at 473, 478, 488, and 493 K showed very poor yields, <5% SA and no 4HBA nor 4HiPh. Reactions at 483 K after 1 h resulted in 48.9% SA and 5.1% 4HiPh, after 2 h in 49.3% SA and 5.3% 4HiPh, and after 3 h in 43.4% SA and 6.3% 4HiPh. These results suggest that the carboxylation of potassium phenoxide does not result in formation of 4HBA, but only SA and 4HiPh, or perhaps that the 4HBA gets carboxylated further into 4HiPh too quickly to be detected. Considering the results reported by Baine et al. [18] and Markovic et al. [13], suggesting that PhOK carboxylation prefers formation of 4HBA over SA, the later suggestion is more likely to be the case; the formed 4HBA gets converted quickly to 4HiPh, which is the reason that it is not being detected in the final product. Furthermore, considering what will later be discussed regarding the degradation of the in situ protonated salt analogues and their degradation above 423 K, it is evident that when using PhOK, less degradation is evident even at higher temperatures (483 K). This could be explained by the lower amount of moisture present in PhOK, which results in a lower effect on in-situ protonation of monocarboxylated salt products to their acid analogues, and their degradation back to phenol. Furthermore, the lack of products after a slight increase of temperature to 493 K could be explained by the narrow range of temperature sensitivity of the decarboxylation reaction. This is supported by the literature, where Gao et. al. [21] suggested that the small presence of water dramatically reduces the activation energy of the decarboxylation reaction of salicylic acid by 25–31%, thus explaining such drastic changes of results obtained even with small differences in temperature.

Figure 1a shows carboxylation of sodium phenoxide, which seems to show more valuable and trending results. The lowest SA yield (12.2%) was obtained at 378 K after 1 h, which with prolonging the reaction time to 2 and 3 h showed increasing trends of SA yields to 19.8 and 28.0%. Similar trends were observed at 393 K and reaction duration of 1, 2, and 3 h, where the obtained SA yields were 23.9, 40.1 and 45.1%, respectively. Reactions carried out at 408 and 423 K and reaction times of 1, 2, and 3 h resulted in plateauing yields of SA, which ranged between 51.9% and 59.9%; slightly lower yields were obtained from reactions with longer reaction times of 3 h, thus suggesting potential degradation, or further upgrading. The lower yields of SA could not be explained with potential consumption since dicarboxylated 4HiPh was not observed. Finally, the reactions with the highest reaction temperature of 448 K showed decreasing trends of SA yields, where after 1, 2, and 3 h of reaction times the obtained yields of SA were 37.3, 44.1, and 17.5%, respectively, thus confirming potential degradation at higher temperatures and longer reaction times. Lastly, all reactions resulted in relatively low amounts of 4HBA, which ranged from a low of 2.1% at 378 K, 1 h up to 10.3% at 423 K, 2 h. It can also be noticed that the reaction with the highest obtained yield of SA is accompanied by the highest yield of 4HBA, and that the reaction with the lowest obtained yield of SA is accompanied by the lowest yield of 4HBA, which would suggest that both products behave in an identical manner, in terms of activation energies, consumption, degradation, etc. 4HiPh was observed only in trace amounts (<1%) in all of the reactions.

### 2.2. Effect of Carbon Dioxide Pressure

**Figure 2:**
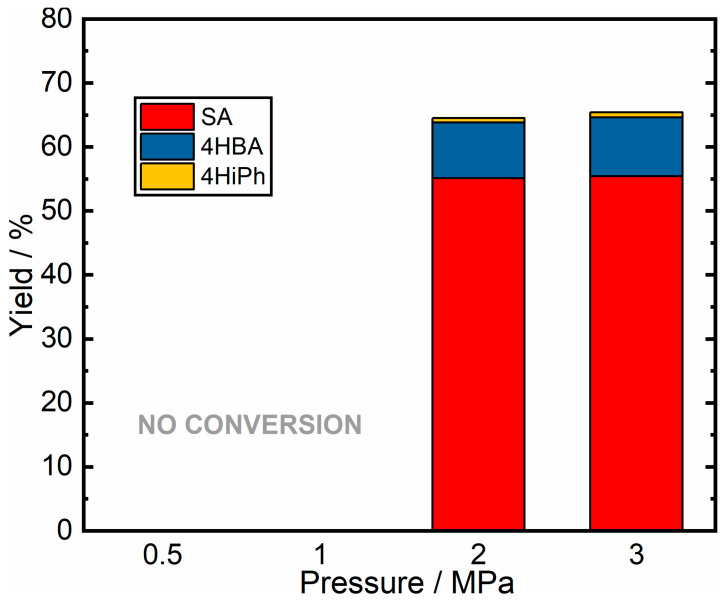
The effect of various CO_2_ pressures (0.5, 1, 2 and 3 MPa) on the conversion of sodium phenoxide and obtained yields of salicylic acid, 4-hydroxybenzoic acid, and 4-hydroxyisophthalic acid (0.3 g PhONa, 408 K, 3 h, 800 rpm).

After investigating the effect of reaction temperature variation on the conversion and selectivity of sodium and potassium phenoxides, it has been observed that sodium phenoxide carboxylation provides more valuable and reliable trends; therefore, it was selected for further investigation of the effect of carbon dioxide pressure. Figure 2 shows reactions carried out at 408 K, 3 h, 0.3 g loading of PhONa, and four different CO_2_ pressures, 0.5, 1, 2 and 3 MPa. Reactions carried out under 0.5 and 1 MPa CO_2_ resulted in no detected products; however, both prepared samples at the end of the reaction accounted for >90% of sodium phenoxide. The missing 10% of carbon balance could be accounted for as char, which was evident as a darker coloration of the solid residue obtained at the end of both reactions. However, reactions carried out under 2 and 3 MPa of CO_2_ showed significant conversion of sodium phenoxide and SA and 4HBA yields. Under 2 MPa of CO_2_ pressure, a 55.1% SA yield was obtained, as well as 8.7% of 4HBA, while under 3 MPa of CO_2_, 55.4% of SA and 9.2% of 4HBA were obtained. Under both reaction conditions, insignificant amounts (<1%) of 4HiPh were observed.

The reason for obtaining such results, where no conversion has been evident under lower pressures (0.5 and 1 MPa CO_2_), could be explained by insufficient amounts of CO_2_ surrounding the molecules of PhONa, and therefore not providing enough contact area and/or inadequate orientation between the two reactants. From simple calculation, considering the room temperature at which the reactor was filled, and reactor headspace of 7 mL, it was calculated that 0.5, 1, 2 and 3 MPa of CO_2_ are equivalent to 1.4, 2.8, 5.7, and 8.6 mmol of CO_2_, while 0.3 g of sodium phenoxide correspond to 2.0 mmol. Therefore, it was concluded that for sufficient conversion of sodium phenoxide, it is necessary to provide at least twice as high a molar concentration of CO_2_, therefore, CO_2_ pressure of at least 2 MPa (under such an experimental setup).

### 2.3. Effect of Metal Phenoxide Loading

First and foremost, the conversion of commercially available metal phenoxides (sodium phenoxide PhONa and potassium phenoxide PhOK) was investigated at elevated temperatures and pressures in a batch regime. This was primarily carried out in a Parr 5000 Multi Reactor System with six 75 mL parallel batch reactors. After conducting the first set of experiments, carried out at 3 MPa of CO_2_ pressure and various temperatures (363–473 K) with 0.3 g (2.0 mmol) of sodium phenoxide for 3 h, it was observed that the yields of salicylic acid (SA), yields of 4hydroxybenzoic acid (4HBA), yields of 4hydroxyisophthalic acid (4HiPh), and conversion of phenol (PhOH) do not show any sensible trends (no sensible trends while varying the reaction temperature to increase any of the observed products). After this observation, it was decided to carry out an additional set of reactions under identical reaction conditions to check for repeatability, and the results obtained show significant deviation from the initially obtained ones. The same approach was applied in a Parr 2500 Reactor system with three 5 mL parallel batch reactors with identical loading of 0.3 g of metal phenoxide (2.0 mmol), and the results were shown to be significantly more repeatable, with lower deviations than those observed in 75 mL reactors. These results suggested that the volume ratio of the metal phenoxide material to the reactor volume, their contact areas, and stirring efficiency, significantly affect this solid-to-gas reaction system. Thereafter, it was decided to proceed with the study using the 5 mL reactor vessels.

**Figure 3:**
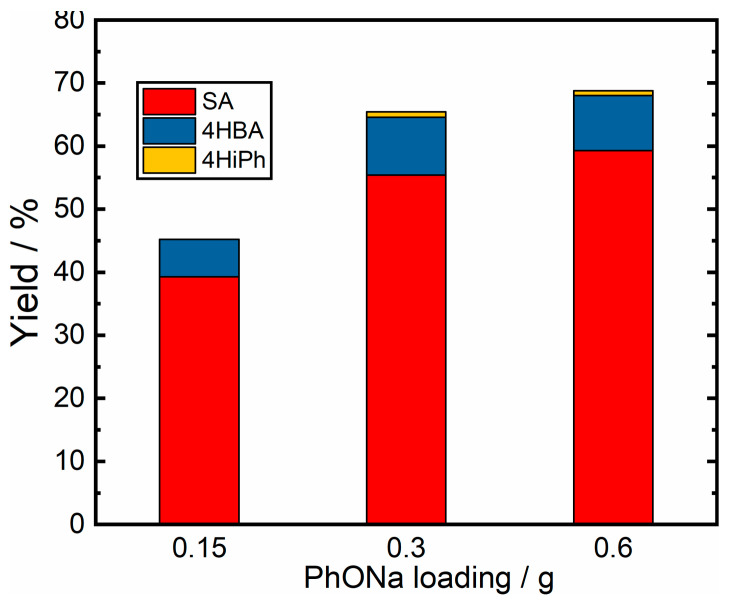
The effect of various metal phenoxide loadings (0.15, 0.3, and 0.6 g) on yields of obtained salicylic acid (SA), 4-hydroxybenzoic acid (4HBA), and 4-hydroxyisophthalic acid (4HiPh) obtained from conversion of sodium phenoxide (408 K, 3 MPa CO_2_, 3 h, 800 rpm).

Furthermore, once the reactor with the lower total volume had been selected as preferable, the effect of sodium phenoxide loading was investigated. As shown in Figure 3, three different amounts of sodium phenoxide were loaded: 0.15, 0.3, and 0.6 g, equivalent to 1.0 mmol, 2.0 mmol, and 4.0 mmol, respectively. The reaction conditions were kept consistent for all three reactions: 408 K, 3 h, 3 MPa CO_2_ pressure, and 800 rpm stirring speed. The lowest loading of 0.15 g resulted in 39.3% SA and 5.9% 4HBA, the medium loading of 0.3 g resulted in 55.4% SA and 9.2% 4HBA, and the highest loading resulted in 59.3% SA and 8.7% HBA. The reaction with the lowest loading also resulted in significant charring of the solid products, which were not dissolved in the solvent mixture, and consequently were not analyzed with HPLC. This observation also explains the lower carbon balance, which for this reaction was 92.9%, while for the reactions with loading of 0.3 and 0.6 g it was close to complete. Although no charring was evident for the latter two reactions, the explanation for their relatively low conversion (57.7 and 65.3%, respectively) could be inefficient stirring, relatively low amounts of CO_2_ available (low pressure) for such high reactant concentrations, and consequently insufficient CO_2_–PhONa contact area. Overall, the results obtained with various loading amounts of sodium phenoxide are still within the range of most other results reported in the literature, carried out in batch reactor systems with gaseous CO_2_ and powdered PhONa [10,18,19]. As such, the ratios of reactor volume 5 mL, magnetic stirring at 800 rpm, 3 MPa of CO_2_, and various loadings of PhONa, ranging between 0.15 and 0.6 g, result in repeatable and substantial yields of salicylic acid (39.3–59.3%) and 4-hydroxybenzoic acid (5.9–9.2%), while there was still no 4-hydroxyisophthalic acid observed.

### 2.4. Reaction Mechanism Determination

**Scheme 1:**
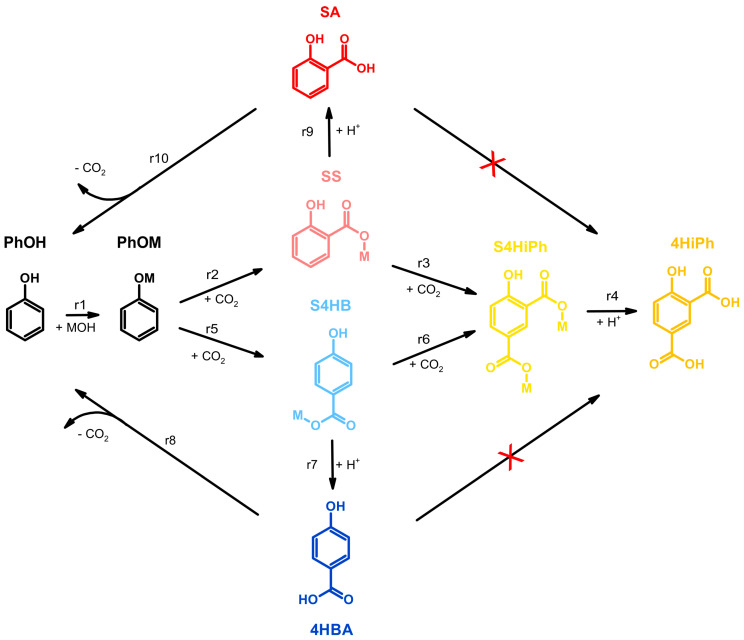
Proposed reaction mechanism of two-step conversion of phenol to mono- and dicarboxylated phenolic derivatives, showing possible obtained products in color, which corresponds to the used color-compound presentation in graphs of observed yields and kinetic model.

The reaction mechanism has already been mentioned in various studies on phenol carboxylation; however, every article still emphasizes the incompleteness, irregularities, and inconsistencies of this rather complex reaction system [13,14,19]. As mentioned above, the complete reaction of phenol carboxylation consists of two steps: the first one, reaction r_1_ from Scheme 1, is the reaction between the phenol and metal hydroxide (KOH, NaOH, LiOH, etc.), or even metal carbonates (K_2_CO_3_, Na_2_CO_3_,etc.). The second step includes a carboxylation reaction under high pressure, high temperature, and preferably dry conditions of the prepared metal phenoxide (PhOM). As discussed in our previous section, metal phenoxide can only be carboxylated by a single CO_2_ unit onto ortho and para positions, forming sodium salicylate (SS) through reaction r_2_, and/or sodium 4-hydroxybenzoate (S4HB) through reaction r_5_. None of the published articles on this topic have ever reported producing 3-hydroxybenzoic acid (3HBA), or its salt derivatives, which was thoroughly explained by Markovic et al. [13].

Moreover, sodium salicylate could be additionally carboxylated, through reaction r_3_, into sodium 4-hydroxyisophthalate (S4HiPh), while sodium 4-hydroxybenzoate, through reaction r_6_, could also be additionally carboxylated into S4HiPh. None of the other combinations of double carboxylated SS and/or S4HB have been detected in any of the conducted experiments, under various reaction conditions, while additionally, no other dicarboxylated phenols have ever been detected and reported as a product of phenol carboxylation reactions. Furthermore, SS, S4HB, and S4HiPh could be converted into their acid derivatives by acidification, as shown in Scheme 1, from reactions r_9_, r_7_, and r_4_, respectively. As debate about the reaction mechanism is still ongoing in the literature, whether the intermediate complex PhOM-CO_2_ is present [22,23] or the direct carboxylation on the benzene ring takes place due to the electrophilic substitution [15], this step is yet to be further investigated. This discrepancy in the reported results, as well as chosen analytical methodology, does not allow us to investigate and observe the presence of either of the mentioned intermediates; therefore, the authors have decided to omit this step, and suggest a single step of carboxylation of PhOM towards either SS (r2) or S4HB (r5), whose kinetic parameters will include the contribution of the intermediate complex PhOM-CO_2_. Further investigations including in situ NMR or FTIR could potentially distinguish the contribution of the additional step and formation of the intermediate complex PhOM-CO_2_ from PhOM and CO_2_.

All of the reactions carried out in our study involved cooling down the reactor vessels, depressurizing the unreacted CO_2_, and dissolving the reaction contents in 5 mmol sulfuric acid, which simultaneously acidified and stabilized synthesized salts, while also preparing the samples for the HPLC analysis. However, when conducting the reactions with already acidified products (commercially available salicylic acid and 4-hydroxybenzoic acid), we did not observe double carboxylated 4HiPh, only phenol (PhOH) and gaseous CO_2_. These reactions were carried out in a nitrogen atmosphere to assure the source of the detected CO_2_ (gaseous sample analyzed with a micro GC), and at various temperatures (393–473 K), which all suggest ready degradation of formed products.

**Figure 4:**
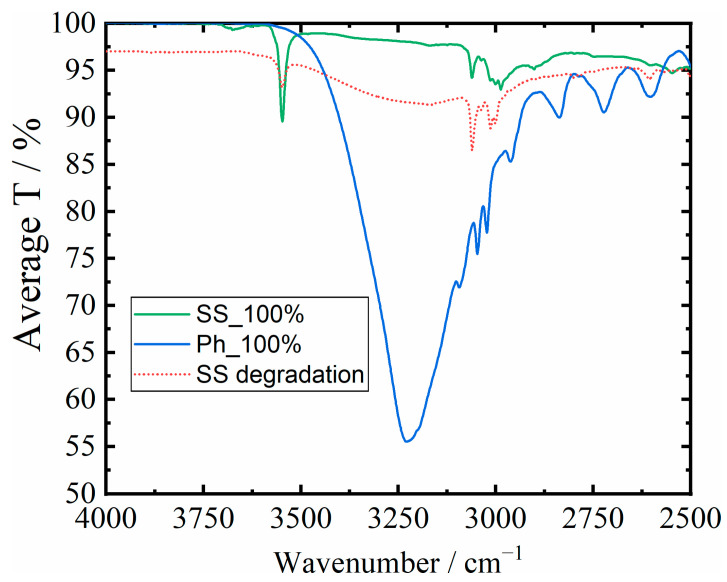
FTIR spectra of pure sodium salicylate (**green**), pure phenol (**blue**), and product of sodium salicylate (**red**) reaction, showing its evident degradation.

Additionally, when starting reactions with their salt analogues, SS and S4HB, under identical reaction conditions, we were able to detect both double carboxylated 4HiPH as well as phenol (PhOH). As shown by the FTIR spectra from Figure 4, green spectra of pure sodium salicylate shows a characteristic peak of −ONa stretching at 3550 cm^−1^, while the pure phenol shows a broad peak between 3500–3100 cm^−1^, characteristic for the −OH stretching. Dotted red spectra, obtained from the sodium salicylate reaction product, directly show a diminishing −ONa peak at 3550 cm^−1^, as well as the appearance of the −OH band at 3500–3100 cm^−1^. The literature also confirms this observation: Hunt et al. [24] showed that monosodium salicylate (SS), when heated to higher temperatures (574 K), yields equimolar amounts of phenol, carbon dioxide and disodium salicylate, while heating sodium phenoxide to 413 K also gives equimolar amounts of free phenol and disodium salicylate, which confirms the possibility of products and intermediates degrading to free phenol. They explain that this takes place due to heterolysis of the anionic form after migration of the metal cation, or even due to the reaction of the metal phenoxide with a second molecule of the metal salicylate to yield phenol and the dimetal salicylate. The latter, however, could not be claimed to take place in our case, since that would suggest the equimolar presence of free phenol and dimetal salicylate (or even higher amounts of dimetal salicylate), whereas in all of our reactions, significantly more free phenol was observed than the dimetal salicylate. Besides the primarily suggested degradation of products and intermediates back to free phenol, the literature [22,24] additionally suggests the presence of thermodynamic equilibrium between phenol and metal salicylate, which results in conversion of only about half of the phenol. This phenomenon was also observed in our study, as confirmed by our results.

The above-mentioned set of experiments allowed us to come to the conclusions that the salt analogues react with the traces of water present in the reactants and/or humidity, which then form the acid derivatives of SS and S4HB—SA and 4HBA, respectively—which can then undergo degradation to gaseous CO_2_ and phenol. These claims have been confirmed by the analysis of the pure sodium phenoxide by thermogravimetric analysis (TGA), as well as with the Karl–Fisher titration method, which was done from multiple measurements, and the results showed the presence of 18.75 and 20.02% of moisture, respectively (shown in Supplementary Materials Tables S1–S4). This set of reactions, for the first time, experimentally proves and explains the notion of equilibrium in phenol carboxylation reactions, the need for and the effect of a dehydrating agent, which would prohibit premature and undesirable water acidification of SS and S4HB, and provide higher yields of mono- and dicarboxylated phenolic compounds obtained from carboxylation of phenol and other potential lignin-derived aromatic compounds.

### 2.5. Kinetic Modelling

**Figure 5:**
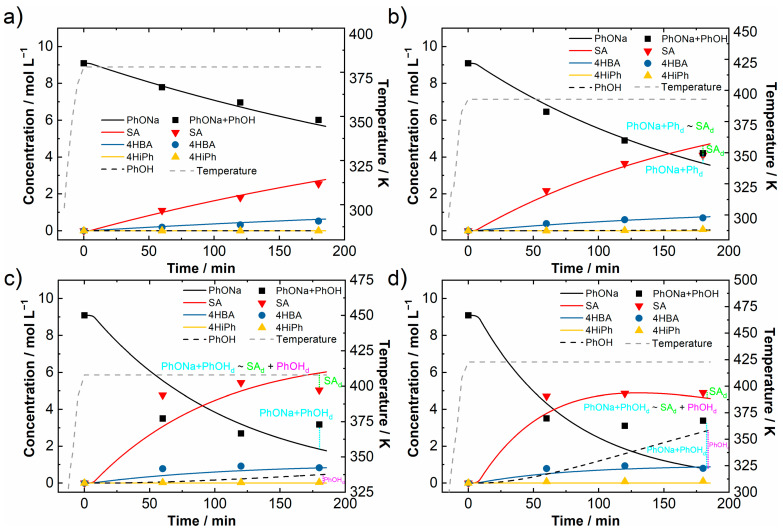
The influence of various reaction temperatures on carboxylation of sodium phenoxide (PhONa) at 378 K (**a**), 393 K (**b**), 408 K (**c**), 423 K (**d**), with experimentally obtained values (**dots**) and modeled values (**lines**).

All the experimentally obtained results reported and discussed above were used in the development of the kinetic model. The experimental results primarily served as a crucial source for the construction of the reaction mechanism, which is shown in detail in Scheme 1. From the reported mechanism, it was possible to describe all the reaction routes (*r_i_*) involving all the specific, experimentally obtained, detected, and quantified products (*j*). The well-constructed experimental plan, which investigated effects of various reaction conditions, successfully complemented the detailed reaction mechanism and allowed for the development of a set of differential equations, which successfully resulted in calculation of kinetic parameters. As shown in Figure 5, it can be seen that the model very well describes the experimentally obtained results. The four graphs each show the reaction temperature: 378 K (Figure 5a), 393 K (Figure 5b), 408 K (Figure 5c) and 423 K (Figure 5d), with a dashed grey line. Each graph also contains experimentally obtained concentrations of each product, shown with scattered dots of various shapes, and modeled concentrations of each product, shown with lines. Modeled and experimentally obtained concentrations of each product have corresponding colors, specific for each product, and matching the colors shown in Scheme 1. Furthermore, the experimental concentrations are obtained from the same reactions as shown in Figure 1; however, here they are represented as concentrations, due to more accurate calculations in the model.

The modeled values very well describe the reactions taking place, and fit well with the experimentally obtained results. It can be seen that with the increase of temperature above 393 K, lower amounts of products (SA and 4HBA) were obtained, which was primarily ascribed to the degradation of the products. In addition to this, as described above and shown in Scheme 1, reactions starting with SA and 4HBA did not yield dicarboxylated product 4HiPh, but pure phenol. These two facts have been incorporated into the model and respective molar balances, and significantly contributed to the accuracy of the modeled values. The results of this finding are seen in the modeled values of pure phenol, which are shown as a dashed black line. As previously discussed, the HPLC methodology does not allow for differentiation between phenol and metal phenoxide; therefore, the unreacted metal phenoxide and phenol (obtained from the degradation of formed products) is accumulatively detected and quantified. However, as shown in Figure 5, the discrepancy in experimentally quantified metal phenoxide (shown with black squares) and the modeled value (shown with a black line) (PhONa + PhOH_d_) corresponds to the modeled value of phenol, shown with a dashed black line (PhOH_d_). These values, when summed and added to the discrepancy of the modeled and experimentally obtained salicylic acid (SA_d_, observed due to degradation of SA), confirm the narrative of degradation of products to phenol, and show great fit of the modeled and experimentally obtained results (Figure S2). In other words, the model successfully calculated the contribution of phenol obtained from degraded products to the experimentally obtained metal phenoxide amounts, which included phenol plus the metal phenoxide.

Lastly, Table 1 shows the values of calculated kinetic parameters. As described above, only protonated compounds were able to be detected and quantified, while the salt analogues could not. Due to this, not all the reactions could have their kinetic parameters calculated accurately; however, the missing ones were described qualitatively. As can be expected, the r_2_ reaction, the formation of sodium salicylate (SS), is faster than the r_3_ reaction, the formation of sodium 4-hydroxybenzoate. Likewise, the degradation of salicylic acid (SA) to phenol through reaction r_8_ is slightly faster than the degradation of 4-hydroxybenzoic acid through reaction r_10_. The protonation reactions (r_7_, r_9_, and r_6_) are considered to be immediate reactions. Reported kinetic parameters are only representative and related to data for the carboxylation reaction of sodium phenoxide, and not other metal phenoxides. This is also evident from the results obtained for the carboxylation of other, non-sodium phenoxides, reported by Rahim et al. [15], where significant deviation of temperatures is required to reach the activation energy and obtain desirable products, i.e., significant difference in temperature–yields correlation is observed when compared to that reported here for sodium phenoxide.

## 3. Discussion

Carboxylation of phenol and similar aromatic reactions are two-step reactions involving an aromatic hydroxide compound, potentially lignin-derived, reacting with a strong base and forming metal phenoxide, which in the second step, under high temperatures and pressures, reacts with CO_2_ to form mono- and dicarboxylated aromatic compounds. Due to the inertness of the CO_2_ compound, stability of the aromatic ring, and its difficult C-H bond activation, this C-C bond formation reaction between aromatic compounds and carbon dioxide is a very challenging procedure. This work provides detailed investigation of the effect of various reaction conditions, involving reaction duration, reaction temperature, reactant loading, and CO_2_ pressure, on the conversion and selectivity of carboxylation of sodium and potassium phenoxides. Potassium phenoxide has been shown to be significantly more sensitive and its meaningful activity was only observed at 483 K, which could be explained with substantial susceptibility to water retention and its consequent degradation, while sodium phenoxide was found to be easily carboxylated at temperatures between 363 and 473 K. Carboxylation of potassium phenoxides carried out between 1 and 3 h yielded salicylic acid (SA) in the range of 43.4 to 49.3%, as well as 4-hydroxyisophthalic acid (4HiPh) in the range of 5.1 to 6.3%. Meanwhile, carboxylation of sodium phenoxide carried out between 1 and 3 h yielded salicylic acid in the range of 11.4 to 47.8%, 4-hydoxybenzoic acid in the range of 2.0 to 8.2%, while the dicarboxylated 4HiPh was only detected in trace amounts. Reactant loading was shown to be directly related with the reactor volume, surface contact area, and efficiency of the stirring, in order to provide efficient mixing and contact between CO_2_ and the phenoxide, as well as to prevent degradation and charring of the products. Finally, CO_2_ pressure was shown to be crucial for the activity and successful conversion of metal phenoxide, where reactions carried out below 2 MPa CO_2_ pressure showed no conversion. Furthermore, the extensive experimental work led to the acquisition of significant amounts of data, allowing the development of a kinetic model which very well describes all the reactions taking place, and provides calculation of kinetic parameters. Lastly, this study has provided a detailed reaction mechanism, with exhaustive experimental data proving potential routes and methods of potential intermediates’ upgrading and/or degradation, with the highest emphasis on water and humidity presence, which can convert newly formed monocarboxylated salts into acidified analogues which easily degrade back to phenol, and thus limit the extent and yields of finally obtained products.

This study was able to, for the first time, accumulate systematic and well-constructed data on the effect of various reaction conditions on the carboxylation of sodium and potassium phenoxides, and development of the kinetic model describing such reactions. As such, it provides valuable information for valorization of other more complex lignocellulosic aromatic compounds and their potential upgrading through C-C bond formation with highly abundant and harmful carbon dioxide.

## 4. Materials and Methods

### 4.1. Materials

All the precursors, reactants and gasses were purchased from commercial suppliers and used as is, without any further purification. Two different commercial metal phenoxides were used in carboxylation reactions: sodium phenoxide (PhONa) (98%, Alfa Aesar, Ward Hill, MA, USA, LOT#: N06G041) and potassium phenoxide (PhOK) (95%, ABCR, Karlsruhe, Germany, LOT#: 1452225). Gaseous carbon dioxide (2.2, Messer, Bad Soden am Taunus, Germany) was used as a reaction gas and gaseous nitrogen (5.0, Messer, Bad Soden am Taunus, Germany) was used for purging the reactor.

For calibration curves used in high-performance liquid chromatography (HPLC), various potential phenolic carbonates were obtained: salicylic acid (SA) (≥99.0%, Sigma-Aldrich, St. Louis, MO, USA, LOT#: MKCK6947), 4-hydroxybenzoic acid (4HBA) (≥99%, Sigma Aldrich, St. Louis, MO, USA, LOT#: BCCB8991), and 4-hydroxyisophthalic acid (4HiPh) (>98.0%, TCI Tokyo Chemical Industry, Tokyo, Japan, LOT#: 7RFGK-MQ). Other mono- (3-hydroxybenzoic acid (3HBA), 99%, Sigma Aldrich, St. Louis, MO, USA, LOT#: BCCB1724), and dicarboxylic (2-hydroxyterephthalic acid (2HtPh) 97%, Sigma Aldrich, St. Louis, MO, USA, 5-hydroxyisophthalic acid (5HiPh) 99%, Acros Organics, Fair Lawn, NJ, USA, and 4-hydroxyphthalic acid (4HPh) >98.0%, TCI Tokyo Chemical Industry, Tokyo, Japan, LOT#: NBX5C-OA) were used in preparation of the appropriate HPLC method and identification of potentially obtained products. However, besides SA, 4HBA, and 2HiPh, no other products were detected, and their calibration curves were not prepared. In-house distilled water was used together with sulfuric acid (95–97%, Honeywell, Charlotte, NC, USA, LOT#: L2490) and acetonitrile (100%, VWR, Radnor, PA, USA, LOT#: 20A094006) for preparation of liquid samples and their HPLC analysis. The used HPLC machine was the

Agilent 1100 (Santa Clara, CA, USA) with the Acclaim Organic Acid column (250 mm length, 4 mm diameter, 5 m particle size) and the pre-column guard cartridge with identical packing (10 mm length, 4 mm diameter, 5 µm particle size). Karl-Fisher titration analysis was done on Metrohm 684 KF Coulometer (Herisau, Switzerland), while TGA analysis was done on TA Instruments TGA Q5000 (New Castle, DE, USA) with 50 mL min^−1^ N_2_ flow and 10 K min^−1^ temperature ramp. The ATR-FTIR analyses were carried out on Spectrum Two Perkin Elmer (Manchester, UK) using a LiTaO3 MIR detector, over the frequency range of 400 to 4000 cm^−1^, with 4 cm^−1^ resolution, 32 scans, and a constant sample pressure of 80 units.

### 4.2. Experimental Reaction Procedures

The desired amount of either sodium phenoxide (PhONa) or potassium phenoxide (PhOK) was introduced into the reactor vessel. Two different reactors were tested. The first was a Parr 5000 Multi Reactor System (Parr Instrument Company, Moline, IL, USA) with six 75 mL parallel batch reactors, individual temperature and pressure controls, and shared magnetic agitation regulator. The second was a Parr 2500 Micro Batch Reactor System (Parr Instrument Company, Moline, IL, USA) with three 5 mL parallel batch reactors, individual pressure controls, and shared temperature and magnetic agitation regulators. Both reactor setups were operated in an identical manner. After introducing the desired amount of metal phenoxide (previously dried, and stored under argon atmosphere to minimize moisture absorption) along with the magnetic bar, the reactor was sealed and agitation was set to 800 min^−1^. The reactor was purged three times with around 1 MPa of N_2_ to expel the air, then three times with around 1 MPa of CO_2_ to expel the N_2_, after which the desired CO_2_ pressure was set. The desired reaction temperature was set with a heat-up rate of 7 K min^−1^, and once the set temperature was reached, the start of the reaction was noted. After the desired reaction duration was reached, the reactors were cooled to room temperature, degassed, and the reaction contents dissolved in 5 mL of 5 mM H_2_SO_4_, filtered and diluted 200 times for high-performance liquid chromatography (HPLC) analysis. Worth noting is that after an observation study of heating sodium phenoxide in an open beaker, it was shown that the matter becomes highly viscous at temperatures significantly below its melting temperature of above 573 K, thus suggesting the presence of the hydrate form of the sodium phenoxide (melting point below 343 K). However, after the TGA analyses (shown in Supplementary Information Figure S1), it was shown that the material only has water physically adsorbed, and not chemically bonded.

The used HPLC machine was an Agilent 1100 with Acclaim Organic Acid column (250 mm length, 4 mm diameter, 5 µm particle size) and a pre-column guard cartridge with identical packing (10 mm length, 4 mm diameter, 5 µm particle size). The constructed method (0.95 mL min^−1^ flow rate, 77.5% 5 mM H_2_SO_4_: 22.5% acetonitrile, 5 µL injection volume, 313 K column temperature) gave great resolution between the peaks of interest. Gaseous products were analyzed with micro GC (Agilent 490 Micro-GC, TCD detectors equipped with PoraPLOT U and CP-COx columns). The obtained concentrations were expressed in terms of conversion of metal phenoxide (PhOM) or as yields of obtained product, for which Equation (1) and Equation (2) were used, respectively.

(1) $$X_{Ph}=\frac{c_{PhOM,0}-c_{PhOM,t}}{c_{PhOM,0}}\times 100\%$$

(2) $$Y_{x}=\frac{c_{x,t}}{c_{PhOM,0}}\times 100\%$$

where:

$c_{PhOM,\;0}$ is the initial concentration of metal phenoxide;
$c_{PhOM,t}$ is the concentration of metal phenoxide at time *t*;
$c_{x,t}$ is the concentration of product *x* obtained at time *t.*

### 4.3. Kinetic Modelling

The model is used to describe the two-phase system, which included the solid/molten reactants and gaseous CO_2_. The reaction mechanism shown in Scheme 1 was used for mathematical calculations of the set of differential equations used for solving all the kinetic rates and concentrations of the analogous compounds. The compounds shown in grey color were not identified (therefore neither were quantified); however, they were kept in the model for more accurate description of the reaction mechanism and its kinetic parameters. For determination and calculation of kinetic parameters, the following assumptions were made:

(1) All the mass of the reactant present in the reactor was considered molten under the reaction parameters and completely available for further reactions.
(2) The availability of CO_2_ to present reactants was considered constant under all reaction conditions and the solubility or solid/gas interaction were not included in the model.
(3) Although the reactor’s shape, size, volume ratio between the surface and reactants volume, and stirrer efficiency were qualitatively shown to affect the reaction, they were not included in the model as the above-mentioned conditions were shown to have repetitive effects on the yields and were considered constant under the tested reaction conditions.
(4) Since the accurate identification and quantification of the products which included unreacted metal phenoxide as well as the resulting phenol was not possible (quantified amounts represent the sum of both), the experimental points were treated as an unreacted metal phenoxide, while the modeled values include calculations of degradation of products and their respective phenol amounts.

Automated constant recording of the temperature and pressure inside the reactor during the entirety of the reaction were used in the Arrhenius equation to describe the reaction rate constants, as shown in Equation (3).

(3) $$k_{i}(T_{2})=k_{i}(T_{1})\times \exp\left(\frac{{Ea}_{i}}{R}\left(\frac{1}{T_{1}}-\frac{1}{T_{2}}\right)\right)$$

The reaction rate (*r_i_*) depends on the reaction rate constant (*k_i_*) and the concentration of the corresponding reactant *j* (*C_j_*), as shown in Equation (4).

(4) $$r_{i}=k_{i}\times C_{j}$$

System of ordinary differential equations were solved numerically in Matlab 2019b software by using ode23tb algorithm.

## Figures and Tables

**Table 1:** Calculated kinetic parameters for sodium phenoxide (PhONa) carboxylation.

i	Product	r_i_	Reaction Rate Constants at 393 K (k_i_)/min^−1^	Activation Energies (Ea_i_)/J mol^−1^
2	SS	*k_2_* ∙ *C_PhOM_*	$4.4\times 10^{-3}$	$5.3\times 10^{4}$
3	S4HiPh	*k_3_* ∙ *C_SS_*	$\ll k_{6}$	N/A
4	4HiPh	*k_4_* ∙ *C_S4HiPh_*	Immediate	N/A
5	S4HB	*k_5_* ∙ *C_PhOM_*	$7.1\times 10^{-4}$	$3.3\times 10^{4}$
6	S4HiPh	*k_6_* ∙ *C_S4HB_*	$\gg k_{3}$	N/A
7	4HBA	*k_7_* ∙ *C_S4HB_*	Immediate	N/A
8	PhOH	*k_8_* ∙ *C_4HBA_*	$8.4\times 10^{-7}$	$1.3\times 10^{4}$
9	SA	*k_9_* ∙ *C_SS_*	Immediate	N/A
10	PhOH	*k_10_* ∙ *C_SA_*	$9.8\times 10^{-5}$	$1.7\times 10^{5}$

## Data Availability

The raw data supporting the conclusions of this article will be made available by the authors on request.

## Supplementary Materials

The following supporting information can be downloaded at: https://www.mdpi.com/article/10.3390/ijms252312923/s1

## References

[1] Tortajada A., Juliá-Hernández F., Börjesson M., Moragas T., Martin R. (2018). Transition-Metal-Catalyzed Carboxylation Reactions with Carbon Dioxide. Angew. Chemie Int. Ed..

[2] Zhang R., Ke Q., Zhang Z., Zhou B., Cui G., Lu H. (2022). Tuning Functionalized Ionic Liquids for CO_2_ Capture. Int. J. Mol. Sci..

[3] Kojcinovic A., Likozar B., Grilc M. (2022). Heterogeneous Catalytic Materials for Carboxylation Reactions with CO_2_ as Reactant. J. CO_2_ Util..

[4] Jasiukaitytė-Grojzdek E., Huš M., Grilc M., Likozar B. (2020). Acid-Catalyzed α-O-4 Aryl-Ether Cleavage Mechanisms in (Aqueous) γ-Valerolactone: Catalytic Depolymerization Reactions of Lignin Model Compound during Organosolv Pretreatment. ACS Sustain. Chem. Eng..

[5] Baral N.R., Banerjee D., Mukhopadhyay A., Simmons B.A., Singer S.W., Scown C.D. (2024). Economic and Environmental Trade-Offs of Simultaneous Sugar and Lignin Utilization for Biobased Fuels and Chemicals. ACS Sustain. Chem. Eng..

[6] Luo J., Larrosa I. (2017). C−H Carboxylation of Aromatic Compounds through CO_2_ Fixation. ChemSusChem.

[7] Park J., Kelly M.A., Kang J.X., Seemakurti S.S., Ramirez J.L., Hatzell M.C., Sievers C., Bommarius A.S. (2021). Production of Active Pharmaceutical Ingredients (APIs) from Lignin-Derived Phenol and Catechol. Green Chem..

[8] Naz S., Bilal A., Saddiq B., Ejaz S., Ali S., Ain Haider S.T., Sardar H., Nasir B., Ahmad I., Tiwari R.K. (2022). Foliar Application of Salicylic Acid Improved Growth, Yield, Quality and Photosynthesis of Pea (*Pisum sativum L*.) by Improving Antioxidant Defense Mechanism under Saline Conditions. Sustainability.

[9] Kolbe H., Lautemann E. (1860). Ueber Die Constitution Und Basicität Der Salicylsäure. Justus Liebigs Ann. Chem..

[10] Lindsey A.S., Jeskey H. (1957). The Kolbe-Schmitt Reaction. Chem. Rev..

[11] Kojčinović A., Likozar B., Grilc M. (2023). Sustainable CO 2 Fixation onto Bio-Based Aromatics. Sustainability.

[12] Cameron D., Jeskey H., Baine O. (1950). The Kolbe-Schmitt Reaction. I. Variations in the Carbonation of p-Cresol. J. Org. Chem..

[13] Marković Z., Engelbrecht J.P., Marković S. (2002). Theoretical Study of the Kolbe-Schmitt Reaction Mechanism. Z. Naturforsch. Sect. A J. Phys. Sci..

[14] Markovic Z., Markovic S., Begovic N. (2006). Influence of Alkali Metal Cations upon the Kolbe-Schmitt Reaction Mechanism. J. Chem. Inf. Model..

[15] Rahim M.A., Matsui Y., Kosugi Y. (2002). Effects of Alkali and Alkaline Earth Metals on the Kolbe-Schmitt Reaction. Bull. Chem. Soc. Jpn..

[16] Plasch K., Hofer G., Keller W., Hay S., Heyes D.J., Dennig A., Glueck S.M., Faber K. (2018). Pressurized CO_2_ as a Carboxylating Agent for the Biocatalytic: Ortho -Carboxylation of Resorcinol. Green Chem..

[17] Sadamitsu Y., Okumura A., Saito K., Yamada T. (2019). Kolbe-Schmitt Type Reaction under Ambient Conditions Mediated by an Organic Base. Chem. Commun..

[18] Baine O., Adamson G.F., Barton J.W., Fitch J.L., Swayampati D.R., Jeskey H. (1954). A Study of the Kolbe-Schmitt Reaction. II. The Carbonation of Phenols. J. Org. Chem..

[19] Luo J., Preciado S., Xie P., Larrosa I. (2016). Carboxylation of Phenols with CO_2_ at Atmospheric Pressure. Chem. A Eur. J..

[20] Iijima T., Yamaguchi T. (2007). The Improved Kolbe-Schmitt Reaction Using Supercritical Carbon Dioxide. Tetrahedron Lett..

[21] Gao L., Hu Y., Zhang H., Liu Y., Song Z., Dai Y. (2016). DFT Computational Study on Decarboxylation Mechanism of Salicylic Acid and Its Derivatives in the Anionic State. J. Mol. Struct..

[22] Marković S., Marković Z., Begović N., Manojlović N. (2007). Mechanism of the Kolbe-Schmitt Reaction with Lithium and Sodium Phenoxides. Russ. J. Phys. Chem. A.

[23] Kosugi Y., Takahashi K. (1998). Carboxylation Reaction with Carbon Dioxide. Mechanistic Studies on the Kolbe-Schmitt Reaction. Stud. Surf. Sci. Catal..

[24] Hunt S.E., Idris Jones J., Lindsey A.S., Killoh D.C., Turner H.S. (1958). 639. Mechanism of the Kolbe–Schmitt reaction. Part II. Influence of the alkali metal. J. Chem. Soc..

