# Supplementary material for: Mechanism, Kinetics and Modelling of Phenol Carboxylation Reactions with CO2
Source: Int J Mol Sci. 2024 Dec 1;25(23):12923. doi: 10.3390/ijms252312923 (PMC11640782; doi:10.3390/ijms252312923)
Supplement: Supplementary file 1 [file ijms-25-12923-s001.zip › ijms-3316296-supplementary.pdf]

Mechanism, kinetics and modelling of phenol carboxylation reactions with CO_2

Aleksa Kojčinović [1,4], Blaž Likozar [1,2,3,*] and Miha Grilc [1,2,4,*]

[1] Department of Catalysis and Chemical Reaction Engineering, National Institute of Chemistry, Hajdrihova 19, 1000 Ljubljana, Slovenia
[2] Pulp and Paper Institute, Bogišićeva 8, 1000 Ljubljana, Slovenia
[3] Faculty of Polymer Technology, Ozare 19, SI-2380 Slovenj Gradec, Slovenia
[4] University of Nova Gorica, Vipavska Cesta 13, 5000 Nova Gorica, Slovenia

---

Supplementary information

---

* Corresponding author. Tel.: +386 1 4760 283; fax: +386 1 4760300.
E-mail address: miha.grilc@ki.si (M. Grilc), blaz.likozar@ki.si (B. Likozar);

Table S1. Prepared sodium phenoxide (PhONa) methanol (MeOH) solution, used for Karl Fisher moisture content measurements.

	PhONa	MeOH	Sum	wt.% PhONa
Weight / g	0.0442	5.8002	5.8444	0.7563

Table S2. Karl-Fisher moisture content measurements of used methanol solvent.

	MeOH			
Aliquot	1	2	3	Average
ppm	285.2	298.4	312.3	298.6

Table S3. Karl-Fisher moisture content measurement of prepared methanol-sodium phenoxide solution.

	MeOH+PhONa	
Aliquot	1	2
Measured moisture content / ppm	1760	1866
MeOH moisture contribution subtracted[1] / ppm	1461.4	1567.4
Moisture in prepared sample[2] / wt.%	0.1463	0.1567
Moisture in original PhNa[3] / wt.%	19.32	20.72

[1] Subtracted the average MeOH moisture content, shown in Table S2
[2] Weight percentage of moisture in prepare sample, as shown in Table S1
[3] Weight percentage of moisture present in the original PhONa, used in preparation of the sample in Table S1, as well as in all the experiments

Table S4. Thermogravimetric analysis (TGA) of used reactant, sodium phenoxide, showing its moisture content.

Sample	1	2	3	Average
Moisture amount / wt.%	19.04	18.52	18.70	18.75

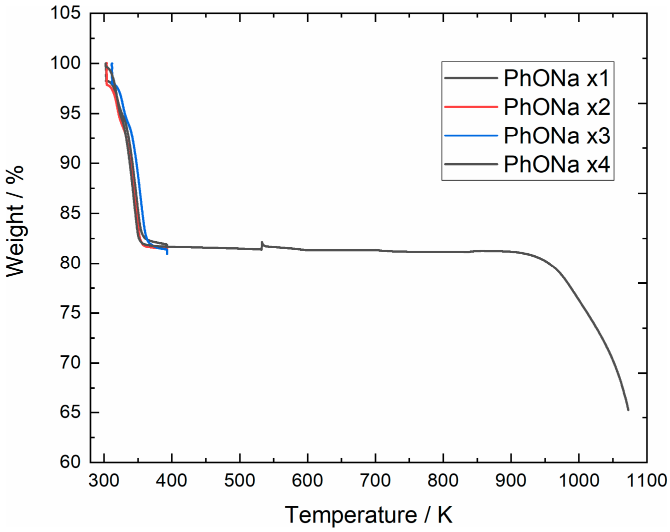

Figure S1. Thermograms of the thermogravimetric analysis of used reactant, sodium phenoxide.

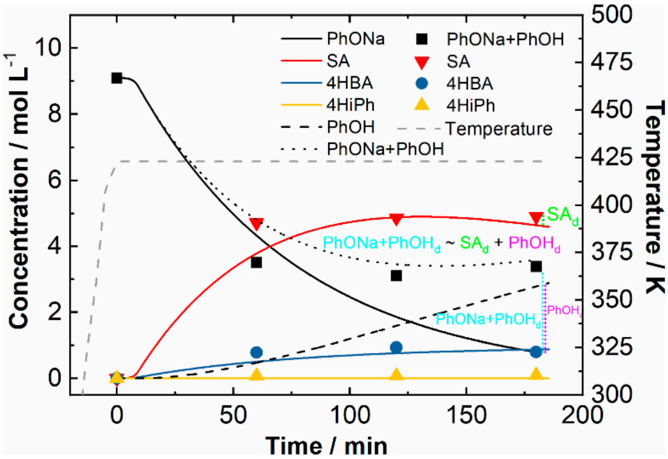

Figure S2. Modeled and experimental values for reaction carried out at 423 K (Fig 5c), showing additional line (black dotted line), including the sum of modeled PhONa and modeled PhOH values.
